# Safety and feasibility of sutureless pars-plana vitrectomy in sub-Tenon anesthesia (SAFE-VISA): a prospective study

**DOI:** 10.1186/s40001-023-01447-2

**Published:** 2023-10-30

**Authors:** Tibor Lohmann, Sabine Baumgarten, Julia Prinz, Peter Walter, Antonis Koutsonas

**Affiliations:** Department of Ophthalmology, RWTH Aachen University, Pauwelsstrasse 30, 52074 Aachen, Germany

**Keywords:** Retina, Retinal surgery, Vitreoretinal surgery, Local anesthesia, Sub-Tenon anesthesia, General anesthesia, Pain, Anxiety

## Abstract

**Background:**

To determine the safety and feasibility of sutureless pars-plana vitrectomy (ppV) in sub-Tenon anesthesia.

**Methods:**

In this prospective study. Pain and anxiety at various times after ppV using a visual analogue scale (VAS) and Wong-Baker-FACES scale as well as visual sensations during surgery were investigated. The surgeon evaluated motility, chemosis, overall feasibility.

**Results:**

ppV was performed on 67 eyes (33 sub-Tenon anesthesia, 34 general anesthesia). Pain during surgery in sub-Tenon anesthesia was 1.8 ± 2.2 (0.0–8.0), anxiety was 2.3 ± 2.2 (0.0–8.5). There was a moderate correlation between pain and anxiety (*R*^2^ = 0.58). Comparing sub-Tenon and general anesthesia no difference in pain perception was found the day after surgery. 27.3% of patients saw details, 21.2% saw colors, 90.1% saw light/motion perception, 3.0% had no light perception. Median chemosis after surgery was 1.0 (IQR = 1.0). Median motility of the eye during surgery was 1.0 (IQR = 1.0), median grade was 1.0 (IQR = 1.0). 24.2% of patients showed subconjunctival hemorrhage during or after surgery.

**Conclusions:**

Sutureless pars-plana vitrectomy in sub-Tenon anesthesia was performed safely, with pain and anxiety levels tolerable for the patients and without the necessity for presence of an anesthesiologist. With 88.9% of patients willing to undergo vitreoretinal surgery in sub-Tenon anesthesia again, we recommend it as a standard option.

*Trial registration* This study was approved by the Institutional Ethical Review Board of the RWTH Aachen University (EK 111/19). This study is listed on clinicaltrials.gov (ClinicalTrials.gov identifier: NCT04257188, February 5th 2020).

## Background

Local anesthesia in ophthalmology dates to the late nineteenth century with Koller and Knapp using cocaine as a topical and retrobulbar anesthetic [1]. With the introduction of advanced amino ester anesthetics such as procaine, lidocaine, bupivacaine or ropivacaine, a wider and safer use for local anesthetics was accessible [2]. Vitreoretinal surgery has formerly been performed under general anesthesia, but local anesthesia has increased in popularity in recent years [2, 3]. In vitreoretinal surgery, topical, retrobulbar, peribulbar and sub-Tenon anesthesia have been described [4].

Topical anesthesia solely using anesthetic eye drops is widely performed in cataract surgery, and its use in vitreoretinal surgery has been shown [5]. Overall, ambiguous results of the efficacy of the anesthesia have been reported and wide-spread adoption did not yet happen [6, 7].

Retrobulbar and peribulbar injections are commonly used, but serious complications have been reported, including brainstem anesthesia with cardiorespiratory arrest, retrobulbar hemorrhage and injury to the optic nerve [8–10]. To minimize these risks, sub-Tenon anesthesia was introduced. After incising the conjunctiva, the anesthetic can be delivered into the sub-Tenon’s space using a blunt cannula. While risk of perforating the globe, injuring the optic nerve, inducing retrobulbar hemorrhage and injecting into intravascularly were reduced, single cases of brainstem anesthesia were reported [2, 11, 12].

The aim of this study was to evaluate the safety and efficacy of single-quadrant sub-Tenon anesthesia without sedation or presence of an anesthesiologist in patients receiving vitreoretinal surgery. Real-life experience in a maximal care hospital indicated that the presence of an anesthesiologist is the bottleneck in surgery planning, not only regarding urgent cases but also planned procedures.

Perceived pain, anxiety and visual sensations were the main outcome on the patients’ side, while the surgeon evaluated eye motility, chemosis and overall surgery performance. Furthermore, the aim was to establish sub-Tenon anesthesia as the standard procedure over general anesthesia for vitreoretinal surgery in cases possible, thus we compared main outcome parameters in sub-Tenon and general anesthesia.

Amidst the course of this study, the corona virus SARS-CoV-2-related global pandemic and resulting cases of COVID-19 respiratory disease created an urgency and demand for the availability of intensive care treatment and artificial respiration, thus leading to reduced capacity of general anesthesia [13, 14].

In an attempt give a methodological quality assessment of local vs. general anesthesia in vitrectomy, Licina et al. concluded that no eligible studies met their inclusion criteria to perform a meta-analysis, thus good-quality clinical trials were needed to define the role of local vs. general anesthesia for pars plana vitrectomy [2]. With the prospective SAFE-VISA study, we supported this cause.

## Methods

### Study type

This prospective single-center study was conducted by the Department of Ophthalmology, RWTH Aachen University.

### Patient selection

**Fig. 1:**
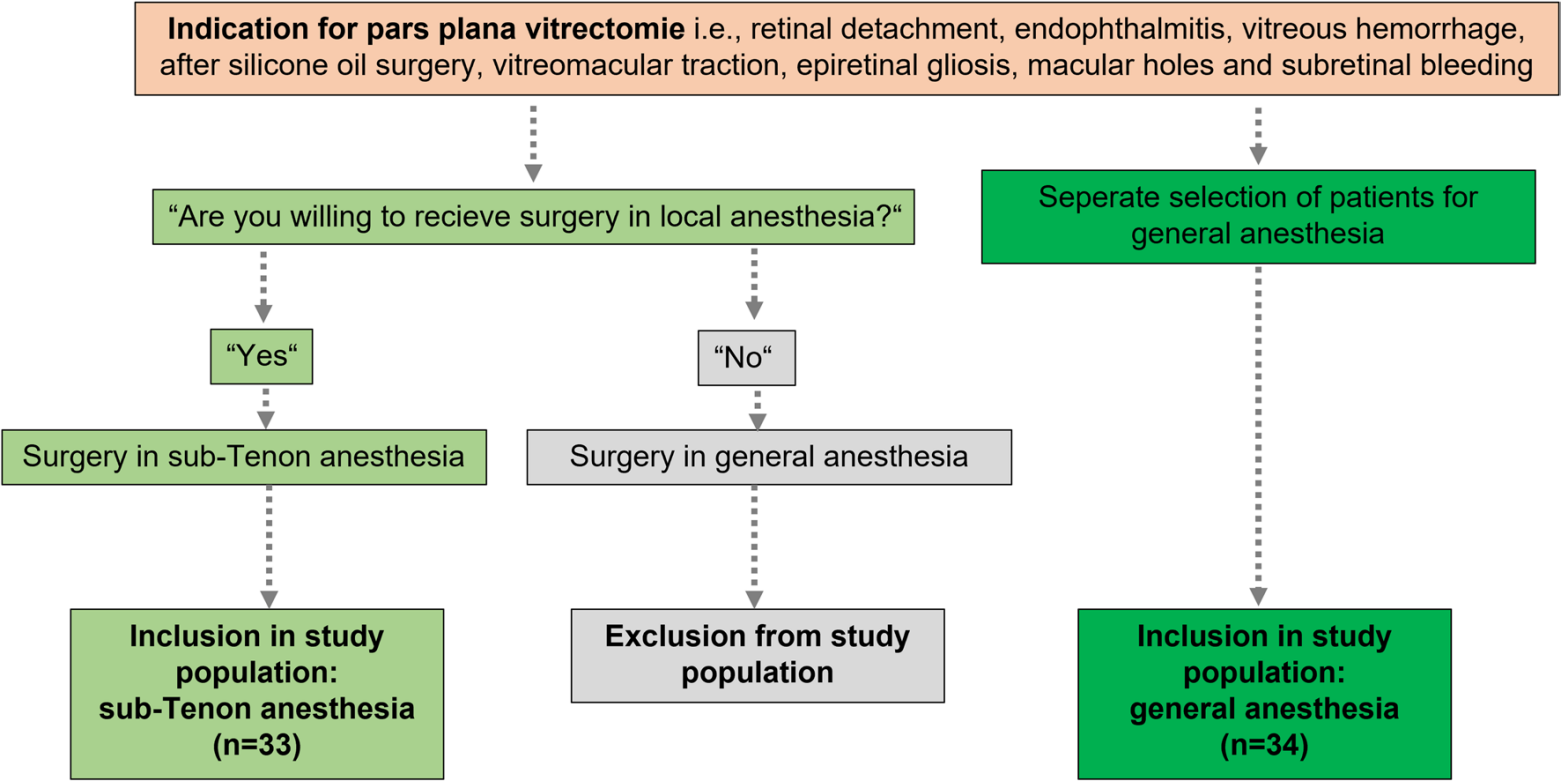
Flow chart of the patient selection process

The study patients undergoing vitreoretinal surgery in the Department of Ophthalmology, RWTH Aachen University, Germany, from November 2019 to December 2021. A patient selection flow chart demonstrates the process (Fig. 1).

### Inclusion criteria

Patients older than 40 years were included with a clinical condition that required surgical treatment with vitrectomy and did not necessarily require general anesthesia (retinal detachment, endophthalmitis, vitreous hemorrhage, after silicone oil surgery, vitreomacular traction, epiretinal gliosis, macular holes and subretinal bleeding).

### Exclusion criteria

Exclusion criteria were concomitant eye diseases such as a state after filtering glaucoma surgery, distinct corneal opacities, complicated proliferative vitreoretinopathy and known allergy to xylocaine and/or bupivacaine. In addition, lack of understanding of the study, its objectives and study conduct, clinically diagnosed psychiatric disorders, pregnancy and simultaneous participation in another clinical trial led to an exclusion. Perioperative anticoagulation for concomitant disease did not lead to an exclusion.

Patients who were initially asked to participate in the study group receiving surgery in sub-Tenon anesthesia, but preferred general anesthesia were not included in the control group to avoid selection bias by over-representing fearful patients in the control group.

### Anesthesia and surgical technique

For sub-Tenon anesthesia, patients received anesthetic eye drops (proxymetacaine–hydrochloride 5.0 mg/ml) three times in 15 min. Disinfection was performed using povidone–iodine (povidone 50.0 mg/ml, iodine 5.5 mg/ml). A conjunctival incision was performed using a conjunctival scissor in the inferonasal conjunctival quadrant. A mixture of 2.0 ml of xylocaine 20 mg/ml and 2.0 ml of bupivacaine 2.5 mg/ml was injected into the sub-Tenon space using a 19 Ga blunt cannula and 2 min of oculopression was applied to spread the local anesthetic. The conjunctival incision did not require sutures.

In the control group, surgery was performed in general anesthesia under supervision of the Department of Anesthesiology, RWTH Aachen University.

For vitreoretinal surgery, patients of both groups underwent a transconjunctival pars plana vitrectomy.

All surgeries were performed by the same surgeon (AK).

Postoperatively, patients received standard topical therapy (prednisolone acetate 10.0 mg/ml eye drops and dexamethasone–dihydrogen–phosphate disodium 1.0 mg/ml and gentamicin–sulfate 5.0 mg/ml eye ointment).

### Data collection

In the study group, a questionnaire was handed to the patients 1 day after surgery. The level of intraoperative pain and pain at the time of answering the questionnaire perceived by each patient were assessed using an 11-point (0.0–10.0) numerical visual analogue scale (VAS). The scale consisted of a linear line subdivided into ten equal intervals, with the leftmost one marked 0.0, indicating no pain, and the rightmost one marked 10.0, representing the worst pain imaginable. In addition, patients were asked to state their pain using the Wong-Baker FACES Scale (FACES). The FACES scale is composed of six black and white cartoon faces ranging from a smiling face representing no pain to a sad, tearful face representing a lot of pain [4] (see also: http://wongbakerfaces.org). Patients were further asked about their level of anxiety during surgery using an 11-point (0.0–10.0) VAS. Finally, patients were asked if they perceived details, colors, light/motion or total darkness during surgery. Questions were answered with “yes”, “no”, or “not sure”.

One day after surgery, the surgeon also received a questionnaire. Presence of chemosis after injection of the sub-Tenon anesthetic and on the day after surgery were answered according to the involvement of conjunctival quadrants (none to four). Globe akinesia was addressed in four possible stages: total akinesia, slight movement, moderate movement, or full movement (none to three). In addition, the surgeon graded every surgery on a scale from one (very convenient, approachable surgery) to six (surgery had to be interrupted several times, additional surgery in sub-Tenon anesthesia was not recommended) like the grading system in German schools.

In the control group, patients received a questionnaire 1 day after surgery. The patients were asked to address pain on the evening after surgery and at the time of answering the questionnaire using the VAS and FACES scale.

### Ethics

This study followed the tenets of the Declaration of Helsinki and was approved by the Institutional Ethical Review Board of the RWTH Aachen University (EK 111/19).

This study is listed on clinicaltrials.gov (ClinicalTrials.gov identifier: NCT04257188).

### Statistics

If not otherwise specified all values were expressed as the mean ± standard deviation (range min–max). Values on the FACES scale were expressed as the median with the interquartile range (IQR). All statistical analyses were performed with GraphPad Prism (GraphPad Prism V7, San Diego, USA). For continuous measures the paired or unpaired *t* test was used in normally distributed data. Wilcoxon signed rank test was used for paired non-parametrical distributed data. Mann–Whitney *U* test was used for unpaired non-parametrical distributed data. Regression analysis was used for detecting possible correlation between perceived pain and surgery duration, patient age or anxiety during surgery. Comparisons between categorical variables were conducted using the Fisher’s exact test. Kolmogorov–Smirnov test was used to identify normal distribution. A *P* value of < 0.05 was considered statistically significant. In cooperation with the Institute of Medical Statistics of the RWTH Aachen University and under consideration of studies and meta-analyses on pain/anxiety assessment in ophthalmological surgeries power calculations yielded in similar group sizes as reported [15–19]. Underlying assumptions from previous studies were based on a discordance rate α of 0.05 and a tolerance probability β of 80.0%.

## Results

The study included 67 eyes of 67 patients undergoing vitreoretinal surgery in the Department of Ophthalmology, RWTH Aachen University, Germany, from November 2019 to December 2021. In 33 eyes vitreoretinal surgery was performed in single-quadrant sub-Tenon anesthesia. The control group consisted of 34 eyes receiving vitreoretinal surgery in general anesthesia. Patients‘ characteristics are displayed in Table 1. Comparing the study and control group, differences were found in patients’ age (*P* = 0.002), surgery duration (*P* = 0.002), and lens status (*P* < 0.001). No differences were found between right or left eye treated (P > 0.999), sex (*P* = 0.305), time between intraocular lens (IOL) implantation and ppV in pseudophakic patients (*P* = 0.961), and visual acuity prior to (*P* = 0.107) and after surgery (*P* = 0.938) (Table 1). Adverse events during follow-up occurred in four cases (12.1%) in the study group, and in three cases (8.8%) in the control group (*P* = 0.709).

**Table 1:** Characteristics of patients who underwent vitreoretinal surgery in sub-Tenon and general anesthesia

Characteristics	Sub-Tenon anesthesia (*N* = 33/67)	General anesthesia (*N* = 34/67)	*P* value
Age (years)	72.9 ± 9.1 (59.0–91.0)	65.4 ± 8.6 (51.0–80.0)	0.002*
Sex			0.305
Male	20 (60.6%)	25 (73.5%)	
Female	13 (39.4%)	9 (26.5%)	
Eye			> 0.999
Right eye	14 (42.4%)	15 (44.1%)	
Left eye	19 (57.6%)	19 (55.9%)	
Lens			< 0.001*
Phakic	1 (3.0%)	13 (38.2%)	
Pseudophakic	32 (97.0%)	21 (61.8%)	
Time between IOL implantation and vitreoretinal surgery (years)	3.7 ± 2.8 (0.3–11.0)	3.3 ± 3.1 (0.5–11.0)	0.961
BCVA (logMAR)			
Pre surgery	1.23 ± 0.92 (NL-0.22)	0.89 ± 0.70 (3.00–0.22)	0.107
Follow-up	0.89 ± 0.95 (NL-0.00)	0.86 ± 1.14 (3.00–0.22)	0.938
Surgery duration (minutes)	22.7 ± 8.6 (12.0–59.0)	32.6 ± 15.1 (13.0–67.0)	0.002*
Indication for surgery			
Rhegmatogenous RD	6 (18.2%)	16 (47.1%)	
Macular hole/Gliosis/VMT	4 (12.1%)	6 (17.6%)	
Silicone oil removal	13 (39.4%)	6 (17.6%)	
Silicone oil removal + peeling	2 (6.1%)	0	
Vitreous hemorrhage	6 (18.2%)	4 (11.8%)	
Endophthalmitis	1 (3.0%)	0	
Sub-ILM hemorrhage	1 (3.0%)	1 (2.9%)	
CRVO	0	1 (2.9%)	
Adverse events during follow-up			0.709
RD	2 (6.1%)	2 (5.9%)	
Vitreous hemorrhage	1 (3.0%)	0	
PVR	1 (3.0%)	1 (2.9%)	

### Sub-Tenon anesthesia

**Fig. 2:**
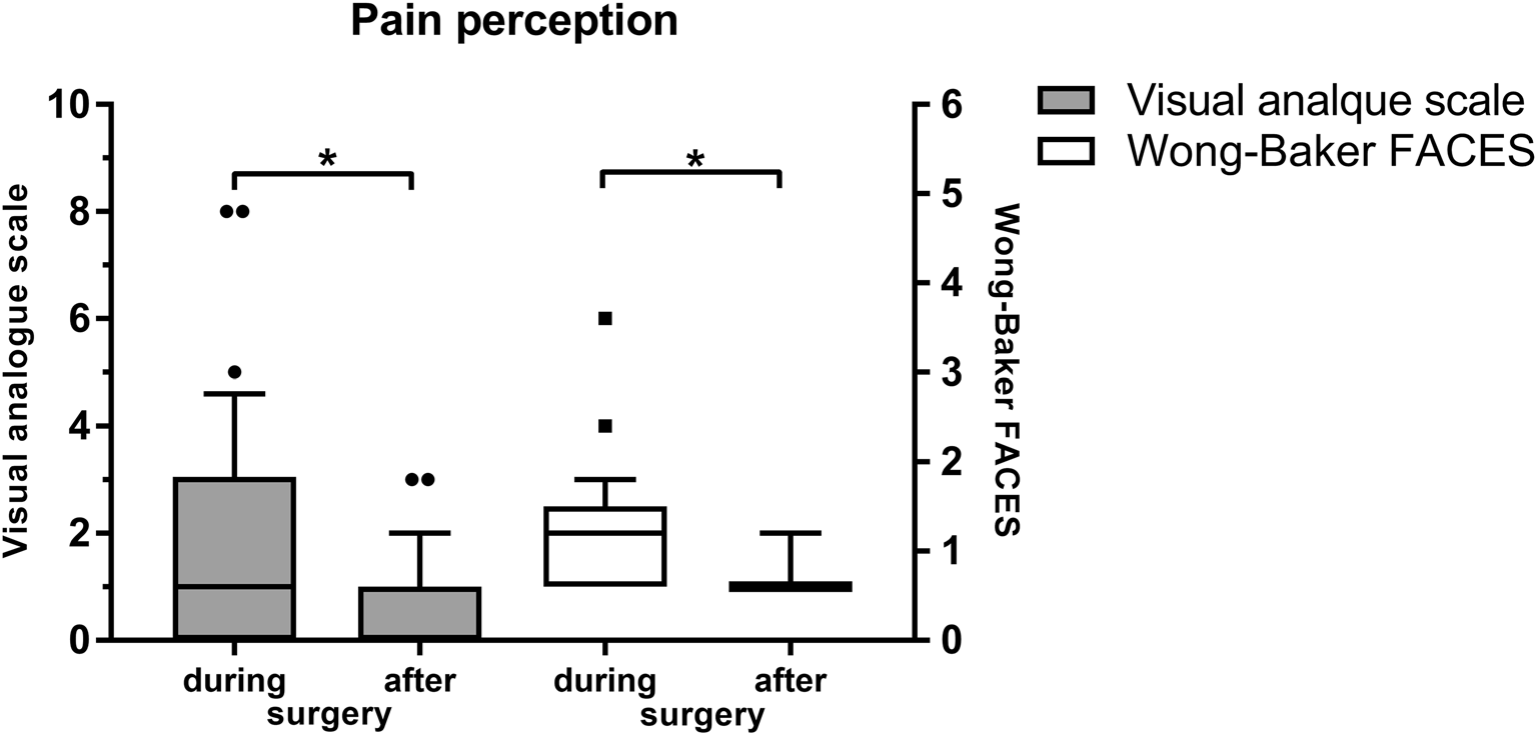
Pain perception during and after vitreoretinal surgery under sub-Tenon anesthesia on the visual analogue scale (VAS) and the Wong-Baker FACES scale. Median is indicated with horizontal line the box. Bottom of the box represents 1st quartile, top 3rd quartile. Whiskers indicate 10th to 90th percentile. Outliers are indicated with staggered black circles and squares, respectively. *P = < 0.05

Perceived pain was higher during surgery compared to the day after surgery, both regarding the VAS (1.8 ± 2.2 (0.0–8.0) vs. 0.5 ± 0.9) (0.0–3.0, *P* = 0.002) and the FACES scale (2.0 (IQR = 1.0) vs. 1.0 (IQR = 0), *P* < 0.001) (Fig. 2). Patients’ mean level of anxiety during surgery was 2.3 ± 2.2 (0.0–8.5) (Fig. 4). A correlation between surgery duration and perceived pain during surgery (*R*^2^ < 0.01) or patient age and pain (*R*^2^ < 0.01) was not found. The level of anxiety on the VAS was 2.3 ± 2.2 (0.0–8.5). We found a moderate correlation between pain and anxiety perceived during surgery (*R*^2^ = 0.58).

After surgery, 27.3% of patients saw details, 21.2% saw colors, 90.1% saw light/motion perception, while 3.0% did not perceive any light during surgery in sub-Tenon anesthesia (Table 2). 88.9% of patients would undergo additional surgery in sub-Tenon anesthesia again.

**Table 2:** Visual perception during vitreoretinal surgery in sub-Tenon anesthesia

	Sub-Tenon anesthesia (*N* = 33)
Details during surgery	8 (24.2%)
Colors	7 (21.2%)
Light/shadow	30 (90.9%)
No light perception	1 (3.0%)

Chemosis improved significantly after surgery (*P* < 0.001) (Table 3). Median motility of the eye during surgery was 1.0 (IQR = 1.0), overall median grading was 1.0 (IQR = 1.0) (Table 3). 24.2% of patients showed subconjunctival hemorrhage during or after surgery.

**Table 3:** Chemosis, Motility and overall grade as evaluated by the surgeon during vitreoretinal surgery in sub-Tenon anesthesia (N = 33)

	Chemosis during surgery (in quadrants affected)	Chemosis say after surgery (in quadrants affected)	Motility during surgery [no motility (0) to total motility (4)]	Overall Grade [without problems (1) to surgery interrupted, difficult (6)]
0	10 (30.3%)	31 (93.9%)	0	–
1	16 (48.5%)	2 (6.1%)	22 (66.6%)	24 (72.7%)
2	5 (15.2%)	0	10 (30.3%)	7 (21.2%)
3	2 (6.1%)	0	1 (3.0%)	2 (6.1%)
4	0	0	0	0
5	–	–	–	0
6	–	–	–	0

All surgical procedures were completed without any adjunctive local anesthesia.

### General anesthesia

**Fig. 3:**
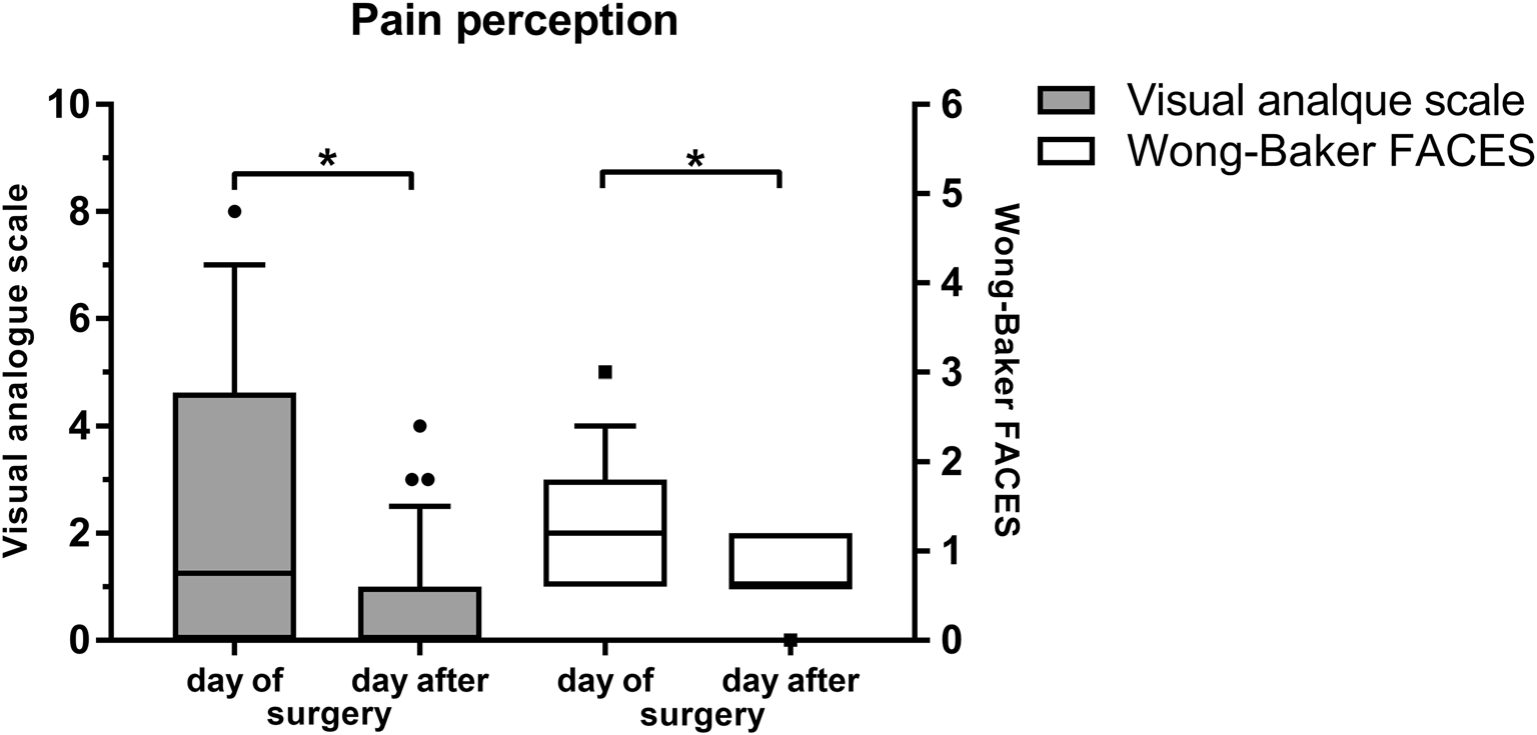
Pain perception on the day of and the day after vitreoretinal surgery under general anesthesia on the visual analogue scale (VAS) and the Wong-Baker FACES scale. Median is indicated with horizontal line the box. Bottom of the box represents 1st quartile, top 3rd quartile. Whiskers indicate 10th to 90th percentile. Outliers are indicated with staggered black circles and squares, respectively. *P = < 0.05

**Fig. 4:**
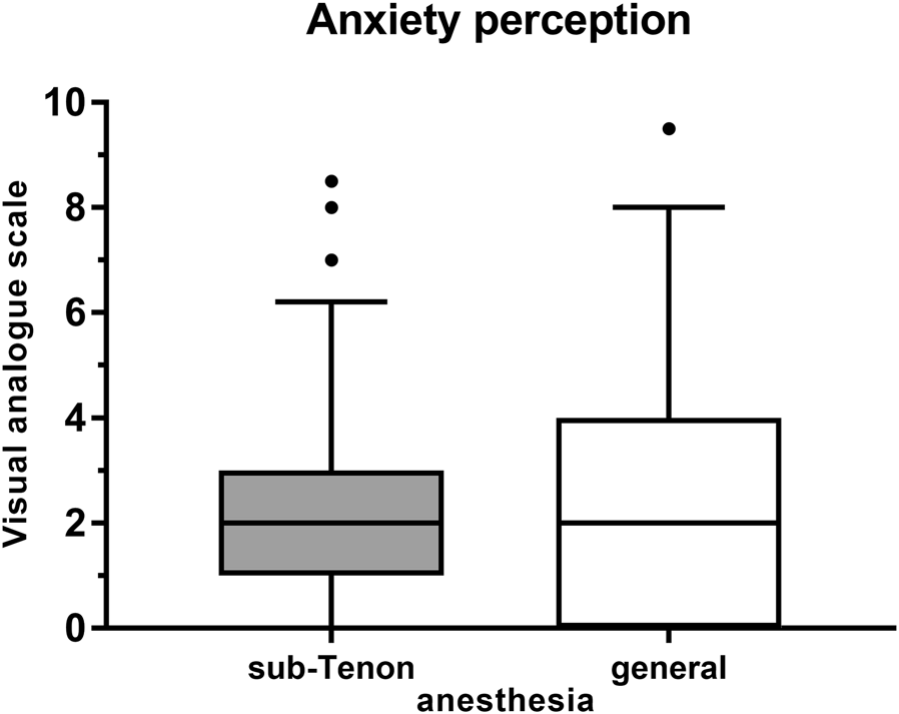
Anxiety perception during vitreoretinal surgery under sub-Tenon anesthesia and prior to vitreoretinal surgery under general surgery on the visual analogue scale (VAS). Median is indicated with horizontal line the box. Bottom of the box represents 1st quartile, top 3rd quartile. Whiskers indicate 10th to 90th percentile. Outliers are indicated with staggered black circles and squares, respectively. *P = < 0.05

In general anesthesia pain on the evening of surgery was higher than on the day after surgery both on the VAS (2.5 ± 2.5 (0.0.–8.0) vs. 0.6 ± 1.0 (0.0–6.0), *P* < 0.001) and on the FACES scale (2.0 (IQR = 2.0) vs. 1.0 (IQR = 0.5), *P* < 0.001) (Fig. 3). Level of anxiety on the VAS was 2.7 ± 2.8 (0.0–9.5) (Fig. 4). Comparing the two, 52.6% of patients stated they were equally anxious about the general anesthesia and the vitreoretinal surgery, while 15.8% were more anxious about the general anesthesia, and 31.6% more anxious about the vitreoretinal surgery.

### Sub-Tenon vs. general anesthesia

Comparing sub-Tenon and general anesthesia no difference in pain perception was found 1 day after surgery regarding the VAS (0.5 ± 0.9 (0.0–3.0) vs. 0.6 ± 1.0 (0.0–4.0), *P* = 0.675) and the FACES scale (1.0 (IQR = 0) vs. 1.0 (IQR = 0.5), *P* = 0.499). During general anesthesia assessment of pain perception was not possible. No difference in pain perception during surgery in sub-Tenon anesthesia and on the evening after surgery in patients receiving surgery in general anesthesia was observed on the VAS (1.8 ± 2.2 (0.0–8.0) vs. 2.5 ± 2.5 (0.0–8.0), *P* = 0.290) and the FACES scale (2.0 (IQR = 1.0) vs. 2.0 (IQR = 2.0), *P* = 0.623).

In sub-Tenon anesthesia, one (3.0%) patient required an analgetic on the day of surgery, while in general, anesthesia seven (20.6%) required an analgetic (five on the day of surgery, two the day after) (*P* = 0.054).

## Discussion

The SAFE-VISA study on safety and feasibility of sutureless vitrectomy in sub-Tenon anesthesia showed good results in terms of intra- and postoperative pain perceived, intraoperative anxiety, intra- and postoperative adverse events, and surgical feasibility. Perceived pain did not differ 1 day after surgery comparing sub-Tenon and general anesthesia. A correlation between perceived pain and surgery duration or pain and patients’ age was not found; none of the patients demanded an anesthetic top up. We found a moderate correlation between levels of pain and anxiety perceived during surgery in sub-Tenon anesthesia.

### Pain

Visual analogue scales have been used successfully to assess pain in various similar studies and in other topics [15, 16, 20, 21].

In a prospective, randomized single center study on 26 patients receiving 23 Ga vitreoretinal surgery for macular hole or epiretinal membrane, Ribeiro et al. reported a median pain score of 1.0 on a VAS from 0.0 to 100.0, with 50.0% of patients reporting no sensation of pain at all during surgery [17]. In their study, patients received 2.0% lidocaine gel in the superior and inferior fornices, followed by a 2.0–4.0 ml 1.0% ropivacaine injection in the sub-Tenon’s space [17]. Furthermore, it is noteworthy that patients in their study received 5.0 ml midazolam 5 mg/ml intravenously [17]. Compared to our study, differences in the applied anesthetics and the standard application of 5.0 ml midazolam 5 mg/ml, a benzodiazepine, could explain the lower reported pain perception. In other settings, a sedoanalgesia combination showed lower levels of perceived pain than analgesia alone [22]. We decided not to use systemic anxiolytic benzodiazepines to reduce risk of cardiovascular depression, avoid obligatory involvement of anesthesiologists and postoperative monitoring, thus reducing workload, and accelerating the workflow up to, and after the surgery itself. 1.0% ropivacaine has been reported to have similar analgetic properties as 0.25% bupivacaine used in our study [23]. Even though surgery duration was longer in their study (62.0 min vs. 22.7 min), pain perception was reported lower than in our study [17]. This supports our finding, that surgery duration is not obligatory correlated with pain experienced.

In a retrospective study on 30 eyes receiving 25 Ga vitreoretinal surgery for macular hole, epiretinal membrane and vitreous hemorrhage for causes other than retinal detachment or proliferative diabetic retinopathy, Roman-Pognuz et al. reported 76.7% of patients perceiving no pain during surgery, and 23.3% perceiving mild pain (2.0 on a VAS from 1.0 to 4.0) [15]. Patients received 5.0 ml 2.0% mepivacaine after topical anesthesia with oxybuprocaine eye drops (concentration not disclosed) three times [15]. In both studies mentioned above, number of patients reporting no perception of pain (50.0% and 76.7%) was lower than in our study (36.3%). Compared to the studies mentioned above, patient age was highest in our study (72.9 vs. 64.0 and 69.6 years), while surgery duration was shortest (22.7 vs. 62.0 and 42.2 min) [15, 17].

In a prospective randomized study, Lai et al. reported pain levels during surgery in sub-Tenon anesthesia of 1.7 on a VAS from 0. to 10.0 in 30 eyes receiving pars-plana vitrectomy (with or without intraocular lens implantation), pars-plana vitrectomy and scleral buckling, or scleral buckling surgery only, matching our results of 1.8 on the VAS [16]. The conjunctiva was opened prior to trocar placement and sutured at the end of surgery [16]. In both our and their study, patients were asked the day after surgery, and curiously, in both studies patients received an anesthetic mixture of 50:50 4.0% lidocaine: 0.75% bupivacaine [16]. While in our study, supplemental anesthesia was not needed, Lai et al. gave 36.7% of the patients a mean 1.6 ml of additional anesthetic mixture [16]. It is of note, that intravenous midazolam (0.5–3.0 mg), fentanyl (20.0–100.0 µg), or propofol (0.0–100.0 mg) was given at a dose determined by the anesthesiologist prior to surgery for sedation [16].

Gill et al. reported pain levels of 3.4 on a VAS from 1.0 to 10.0 intraoperatively [18]. In their prospective study, 27 patients received a single 5.0 ml inferonasal sub-Tenon injection of a 50:50 mixture of 2.0% lidocaine and 0.5% bupivacaine with 150.0 IU hyaluronidase, an enzyme suspected to reduce the effective anesthetic volume [18, 24]. Oculopression after application of the sub-Tenon anesthesia was not applied [18]. Patients did not receive any additional sedatives [18]. 70.4% of the patients received cryotherapy, while none of our patients underwent that treatment, potentially causing a higher pain perception [18].

Regarding pain perceived during surgery, all authors concluded, that sub-Tenon anesthesia is a valid option, similarly effective as retrobulbar anesthesia, and more effective than peribulbar anesthesia [4, 15, 17, 22]. Gill et al. added that a two-quadrant sub-Tenon injection provided significantly better perioperative anesthesia for vitrectomy compared with a standard single-quadrant technique using the same mixture [18].

Bayerl et al. compared pain perception after 23 Ga vitrectomy under general anesthesia with and without additional retrobulbar anesthesia in 130 eyes in a prospective setting [25]. Twenty-four hours after surgery, only one patient (2.4%) receiving sub-Tenon anesthesia only reported pain over 2.0 on a numerical pain scale, similar to the VAS used in our study [25]. In our study, 8.8% of patients had pain levels above 2.0 on the VAS and mean perceived pain of 0.6. The authors concluded that additional retrobulbar anesthesia was not beneficial in preventing or reducing pain [25].

A patient acceptable symptomatic state (PASS) is understood as the outcome score on the VAS a patient needs to have (or better) to “feel good”, and is defined, i.e., for various orthopedic diseases [26]. For vitreoretinal surgery, or ophthalmological surgery in general, a PASS has not been defined yet.

### Anxiety

To our knowledge, none of the studies published on sub-Tenon anesthesia for vitreoretinal surgery incorporated a VAS for anxiety. The VAS to evaluate anxiety has been validated [27]. We showed a moderate correlation between pain perception and anxiety during surgery, fortifying the need for adequate analgesia. In a systemic meta-analysis by Obuchowska et al. on anxiety and fear in cataract surgery, pain during surgery was identified to be the second most common cause of anxiety (41.0%) [28]. They concluded, that next to preoperative education and counselling for patients sufficient analgesia is crucial to reduce anxiety and fear [28]. While reports on anxiety perception during cataract surgery are scarce, Foggitt et al. reported a median anxiety level of 2.0 of 7.0 on the VAS in 108 patients receiving, higher than the 2.3 of 10.0 during surgery we found [29]. Overall, they deemed anxiety levels detected to be acceptable for surgery in local anesthesia [29].

### Visual sensation

In a prospective questionnaire survey, Vohra et al. reported that 90.0% of patients perceived light at some stage during vitreoretinal surgery under local anesthesia [4]. Of these, 70.8% observed movements, 62.5% saw colors, 52.8% saw instruments and 33.3% saw flashes. The commonest observations were colorful swirls, black pipes, and the color red [30]. 77.5% of patients received sub-Tenon anesthesia, while the rest received peribulbar block [30]. Interestingly, 10.0% of patients reported to have not experienced any sensation of light during the entirety of the procedure, while, in our study only 3.0% stated to not have seen any light [30]. This difference could be explained by 12.5% of patients having received peribulbar block as anesthesia [30]. In other studies, a higher percentage of patients not perceiving any light was reported, too [31]. Here, the difference could be explained due to patients being asked about their visual sensations during surgery, and not the day after [31]. 2.7% of their patients felt that the experienced light perception was “frightening”, while the rest deemed it to be either “pleasant” (22.2%) or “bearable” (72.2%) [30]. In our study, mean level of anxiety during surgery on the VAS was 1.3 in patients who reported to see details during surgery, while patients not seeing details reported an anxiety level of 2.8 (*P* = 0.069). Overall, Vohra et al. and our study did not find evidence that visual perception during surgery is linked to a disadvantageous course of the surgery [30].

### Motility

Roman-Pognuz et al. established a score system evaluating motility in each of the four rectus muscles and adding it up to form a global motility score. 13.3% of patients receiving sub-Tenon anesthesia showed absolute akinesia (sum score of 0.0) 5 min after anesthetic application, and 26.7% another 5 min later [15]. In our study, none of the eyes were evaluated as being totally akinetic during surgery, while 63.6% had slight residual motility. Gill et al. reported total eyelid akinesia in 11.1% of patients, 51.9% with partial function and 37.0% with full kinetic function of the eyelids [18]. In general, both studies conclude that sufficient akinesia was achieved in sub-Tenon anesthesia to perform vitreoretinal surgery safely.

### Complications

Subconjunctival hemorrhage occurred in 24.2% of patients receiving sub-Tenon anesthesia. Seen as a minor complication, patients, especially when using antithrombotic agents, should be informed about the transient and innocuous character of the bleeding [32, 33].

Gill et al. reported on chemosis, a parameter we also incorporated in our study. 0.6 quadrants were affected in their group of patients, compared to 1.0 quadrants in our study [18]. There was no information on the course of chemosis on the day after surgery. Overall, chemosis did not affect the surgery. The incidence of chemosis in general is variable and depends on length of cannula, volume of the anesthetic, speed of injection and entry to the sub-Tenon’s space [33]. Lerch et al. reported 14.8% of patients had chemosis that affected one quadrant, and 4.5% of eyes had chemosis affecting two or more quadrants [34]. In our study, 45.5% of patients had chemosis in one quadrant, 21.2% in two or more quadrants during surgery. Differences in the cannula (single-use sterile polyurethane vs. metal blunt cannula in our study) used and speed of application could have led to higher rates of chemosis in our study. In our study, patients with filtrating glaucoma surgery in the past were excluded. In these patients, chemosis must be taken seriously [33].

Slight and severe complications such as cilioretinal artery occlusion, anaphylaxis, perforating the globe, injuring the optic nerve, inducing retrobulbar hemorrhage and injecting intravascularly resulting in brainstem anesthesia were not seen [2, 11, 12, 33, 35]. We conclude that sub-Tenon anesthesia can be applied safely in vitreoretinal surgery.

### Limitations

The design of the study made it impossible to mask the groups or blind the surgeon. Randomization of both study arms was not practical, since in Germany, vitreoretinal surgery in general anesthesia is the standard procedure. As described above, we intentionally did not include retrobulbar anesthesia in our study, due to a reported increased risk of complications. Other studies did not show inferiority of sub-Tenon anesthesia [15–17]. While asserting world-wide use of sub-Tenon anesthesia is difficult, especially in vitreoretinal surgery, there seems to be a move away from retro/peribulbar anesthesia using a sharp needle, towards sub-Tenon or topical anesthesia in cataract surgery [33]. As this study was non-randomized by design, the possibility of confounding factors must not be overlooked. Patients who denied surgery in sub-Tenon anesthesia were not included in the group of patients who underwent surgery in general anesthesia. In doing so, possibly overly sensitive patients were not over-represented in the respective group. As for patient characteristics, patients in the sub-Tenon group were older (*P* = 0.002) and more often pseudophakic (*P* < 0.001). While no conclusive statements were made, studies on the perception of pain in relation to age suggested that older patients report lower intensity of postoperative pain, though not specifically in vitreoretinal surgery [36]. Considering lens status, studies on pain perception during surgery on the first compared to the second eye should be taken into account [19]. For second eye surgery, higher pain perception was reported, possibly related to lower anxiety before the second surgery [19]. Pain and anxiety were enquired on the day after surgery. While different results immediately after surgery were possible, assessing these parameters on the day after surgery is common practice [37]. A significant difference in surgery duration reflects the real-life data the population was drawn from. It could also be the case, that in general anesthesia, the surgeon felt more comfortable with taking time during surgery. Further studies could investigate differences in sub-Tenon surgery with and without sedoanalgesia or an anesthesiologist in stand-by, respectively.

## Conclusions

Our study was able to show that vitreoretinal surgery for various indications can be performed in sub-Tenon anesthesia safely, with pain and anxiety levels tolerable for the patients and without the necessity of the presence of an anesthesiologist. We found a correlation between pain and anxiety perception during sub-Tenon anesthesia highlighting the importance of sufficient anesthesia. With 88.9% of patients willing to undergo vitreoretinal surgery in sub-Tenon anesthesia again, we feel comfortable to offer surgery under local anesthesia as a standard option when possible.

## Statistics

All statistical analyses were performed with GraphPad Prism (GraphPad Prism V7, San Diego, USA).

## Data Availability

The data sets used and/or analysed during the current study are available from the corresponding author on request.

## Abbreviations

SAFE-VISA: Safety and feasibility of sutureless pars-plana vitrectomy in sub-Tenon anesthesia
VAS: Visual analogue scale
FACES: Wong-Baker-FACES-scale
IQR: Interquartile range
IOL: Intraocular lens
ppV: Pars-plana vitrectomy
PASS: Patient acceptable symptomatic state
